# A Systematic Review of Parent–Child Communication Measures: Instruments and Their Psychometric Properties

**DOI:** 10.1007/s10567-022-00414-3

**Published:** 2022-09-27

**Authors:** Holger Zapf, Johannes Boettcher, Yngvild Haukeland, Stian Orm, Sarah Coslar, Silke Wiegand-Grefe, Krister Fjermestad

**Affiliations:** 1 Department of Child and Adolescent Psychiatry, Psychotherapy and Psychosomatics, University Medical Center Hamburg-Eppendorf, Martinistrasse 52, 20246 Hamburg, Germany; 2 Department of Psychology, University of Oslo, Oslo, Norway; 3 Division Mental Health Care, Innlandet Hospital Trust, Brumunddal, Norway; 4 Faculty of Behavioral and Social Sciences, University of Groningen, Groningen, The Netherlands

**Keywords:** Parent–child communication, Adolescents, Parents, Systematic review

## Abstract

Parent–child communication represents an important variable in clinical child and family psychology due to its association with a variety of psychosocial outcomes. To give an overview of instruments designed to measure the quality of parent–child communication from the child’s (8–21 years) perspective and to assess the psychometric quality of these instruments, we performed a systematic literature search in Medline and PsycInfo (last: February 25, 2022). Peer-reviewed journal articles published in English with a child-rated instrument measuring the quality of parent–child communication were included. Initial screening for eligibility and inclusion, subsequent data extraction, and quality assessment were conducted by couples of review team members. Based on the screening of 5115 articles, 106 studies reported in 126 papers were included. We identified 12 parent–child communication instruments across the studies. The Parent-Adolescent Communication Scale (PACS) was used in 75% of the studies. On average, the evidence for psychometric quality of the instruments was low. Few instruments were used in clinical and at-risk samples. Several instruments are available to rate parent–child communication from the child’s perspective. However, their psychometric evidence is limited and the theoretical foundation is largely undocumented. This review has limitations with regard to selection criteria and language bias.

*Registration* PROSPERO: CRD42021255264.

**Supplementary Information:**

The online version contains supplementary material available at 10.1007/s10567-022-00414-3.

Parent–child communication is a fundamental component of family functioning, both from an empirical (e.g., Liu, 2003; Ochoa et al., 2007) and a conceptual perspective (e.g., Estlein, 2021; Papini et al., 1990; Stamp, 2004). Even before a child is born, parents respond to the child’s signals from the womb. This communication marks the start of an enduring interactional process in which children and parents mutually influence each other to create a relational bond that constitutes the child’s inner working model for social relations (Dixson, 1995). The quality of the parent–child communication has been found to influence multiple psychosocial outcomes. At the child level, these factors include socio-relational factors such as peer competence and conflict management (Branje, 2008; Carson et al., 1999), academic factors such as school readiness and performance (Noller & Feeney, 2004), socio-cognitive factors such as moral reasoning, self-esteem, self-development, and individuation (e.g., Arnett, 1999; Grotevant, 2001; McLean et al., 2007), resiliency, and happiness (e.g., Fitzpatrick & Koerner, 2005; Jackson et al., 1998); as well as psychosocial adjustment and mental health (e.g., Davidson & Cardemil, 2009; Houck et al., 2007; Park & Koo, 2009). There is also evidence of longitudinal effects, with a study showing that lack of parent–child communication at age 10 years predicted depression 20 years later (Lindeloew, 1999). Since parent–child communication influences these outcomes from birth to young adulthood, we use the term “child” in the current review with reference to the relationship to the parent, not to a specific age or developmental stage.

At the family level, factors associated with parent–child communication include family relationship quality (Barnes & Olson, 1985), family functioning, adaptability, and cohesion (Koerner & Fitzpatrick, 2002; Schrodt, 2005; Sillars et al., 2014), family satisfaction (Jackson et al., 1998), conflict avoidance (Koerner & Fitzpatrick, 1997), reticence (Kelly et al., 2002), and problem-solving (Olson et al., 1979). Whereas some of the relations between parent–child communication and other variables are assumed to be direct, parent–child communication is also proposed as a potential intermediate variable in predicting child mental health from other variables, such as maternal depressive symptoms (McCarty et al., 2003). Given the importance of parent–child communication for child outcomes, the field needs high-quality parent–child communication measures. The current study aims to provide a quality-based review of such measures.

In determining the optimal ways to measure parent–child communication, multiple methodological issues need to be considered. The first relates to how parent–child communication is conceptualized. The term “communication” represents a wide and varied construct that is difficult to define comprehensively across theories (Krauss & Fussell, 1996). Interpersonal communication composes both speech and non-speech-message aspects and includes a focus on interaction patterns and difficulties, social support, verbal confirmation, boundary management, speech accommodation, self-disclosure, nonverbal cues, and secrets (Vangelisti & Caughlin, 1997). As far as interpersonal communication in the family is concerned, it usually comprises verbal and nonverbal two-way interactions that express feelings, thoughts, values, and needs (Satir, 1988). This basic definition is the basis for multiple operational definitions. Parent–child communication has been conceptualized as an indicator of relationship quality (Huizinga et al., 2005), but also as a routine interaction that defines and shapes parent–child relationships (Dixson, 1995). Because parent–child communication is tightly associated with other psychosocial measures, some researchers may choose to examine parent–child communication through related terms such as relationship quality, attachment, or parenting styles (Feddern Donbaek & Elklit, 2014; Moilanen et al., 2018). The field needs clarification concerning what should be considered the core components of parent–child communication.

A second and related methodological issue is the theoretical basis for parent–child communication. Parent–child communication can be placed in multiple theoretical frameworks, such as social learning theory, attachment theory, family systems theory, role theory, and family process theory (Stamp, 2004). Theoretical plurality is beneficial to the field, and theory development is a constant process within child and family psychology. At the same time, increased awareness of the theoretical background of existing measures would help to bring clarity to the field and provide directions for future research and theory development.

A third methodological issue when considering how to measure parent–child communication is whose perspective this variable should be measured from. At least three perspectives are relevant, i.e., the child, the parent, and potential observers. Empirical knowledge indicates that these perspectives tend to be moderately correlated at best (e.g., Guilamo-Ramos et al., 2006; Hartos & Power, 2000a, 2000b; Hadley et al., 2013). This does not imply that one perspective is more “correct” than the other, but rather reflects the fact that parent–child communication, like many other child psychosocial variables (e.g., mental health symptoms), looks different from different viewpoints (De los Reyes & Kazdin, 2005). This phenomenon is linked to attribution theory and actor-observer differences and should not be considered measurement error (De los Reyes & Kazdin, 2005). However, it is evident that practitioners and researchers need to carefully consider whose rater perspective is optimal for the concept they aim to examine. For example, measuring parent–child communication from an observer’s perspective may be useful if the aim is to identify objectively measurable communication components such as eye contact, gestures, and voice pitch. In contrast, the parental perspective may be of special interest if the parent’s perception of parent–child communication is assumed to relate to parental mental health. However, if the main aim is to examine how parent–child communication is related to the child’s psychosocial functioning, the child’s own perspective may be most useful due to common-rater variance (Achenbach et al., 1987). In the current review, we focus on child-rated measures. There are five main reasons for this. The first reason is related to theoretical perspectives concerning parent–child communication rater overlap. The generational stake theory (Acock & Bengtson, 1980) suggests that parents and children have different psychological needs and different investments in establishing the generational bond due to representing contrasting generations. Whereas parents may invest more in maximizing and maintaining intergenerational continuity, children may be more prone to seek separate identities and therefore emphasize and exaggerate conflicts and differences with parents more. Based on this theoretical perspective, focusing on the child perspective on parent–child communication may be particularly important. A second reason to focus on child-rated measures is that children’s subjective communication experience is likely to be more relevant to assess family functioning and other child-related psychosocial variables (Kapetanovic & Boson, 2022; Xiao et al., 2011). Third, the child perspective may also be more relevant for child-focused intervention planning, as observer-rated data may not converge with how family members assess the situation (Noller & Feeney, 2004). Reviews have shown that the child’s own perspective and children’s active involvement in research about their psychosocial situation is largely under-utilized (e.g., Facca et al., 2020; Larsson et al., 2018). Hence, a fourth reason to focus on child-rated measures is that this may promote the use of children as informants in research. The final reason relates to relevance for the practice field. Self-report questionnaires are more accessible and less resource-demanding to administer than observer-rated measures. Providing an overview of easy-to-administer child-rated measures will thus have high relevance for the practice field.

A fourth methodological issue to consider is the scope or focus of the parent–child communication measure. Definitions of parent–child communication are wide and varied (Vangelisti & Caughlin, 1997), which opens up several measurement angles. Measures can be focused on topics [e.g., sexuality (Sales et al., 2008), health behavior (Miller-Day & Kam, 2010), conflicts (Peterson, 1990)], and/or situations/settings [e.g., home, laboratory (Hadley et al., 2013)], and/or refer to the general quality of parent–child communication (Barnes & Olson, 1982). In addition, measures can address dyadic communication between the child and one parent or triadic communication between the child and both parents. Furthermore, measures can focus on communication quality, frequency, or a combination of these (Miller-Day & Kam, 2010; Xin et al., 2021). A related issue is the time perspective of the measure, i.e., concurrent, prospective, or retrospective. In the current review, our interest lies in measures of parent–child communication that are widely applicable, especially with regard to child mental health and development. Therefore, we focus on the quality of current general parent–child communication and, if subscales are provided, their specific features.

Finally, measures can be tailored for different populations. Whereas some measures are meant for the general population, others are tailored for ethnic groups, nationalities, or age groups. In the current review, we focus on measures for the general child population that can be applied to clinical and at-risk populations as well. “Clinical” indicates that the child has been diagnosed with a mental health or somatic disorder, whereas “at-risk” indicates that the sample was selected according to criteria that are considered as a transitory or continuous risk for child mental health such as being a minority or being bereaved.

The aim of the current study is to provide the field with an overview of existing instruments that measure the quality of parent–child communication from the child’s (8–21 years) perspective. We will consider the psychometric properties of the scales using criteria based on De los Reyes and Langer (2018). We investigated the following research questions: Which child-report questionnaires exist to measure parent–child communication, what kind of samples have they been applied to, and what is their psychometric quality? We will also consider the instruments’ availability, including translations and norms, to ease the decision-making processes for practitioners and researchers who aim to measure parent–child communication.

## Methods

We conducted this systematic review according to the Preferred Reporting Items for Systematic Reviews and Meta-Analyses (PRISMA) guidelines (Page et al., 2021). We searched the PROSPERO database initially to ensure that no similar studies had been started or planned and published a protocol for this study under the number: CRD42021255264.

### Eligibility Criteria

The eligibility criteria were as follows: original, peer-reviewed journal articles published in English language and assessing the quality of general communication between parents and their children via multi-item scales for child self-report were included. In the context of this review, communication included verbal, nonverbal, cognitive, and affective aspects of the interaction between parents and their children, but not the physical ability to communicate. Studies assessing broader concepts such as general family communication or studies using single questions or ad-hoc measures to assess parent–child communication were excluded, just as studies on specific topics of communication such as health-related behaviors (e.g., sex, alcohol, tobacco use). Studies reporting only parent ratings were excluded.

The age range of the study population was set at 8 to 21 years of age, including older children, adolescents, and emerging adults. We included studies examining parent–child communication in general, clinical (both somatic and mental health), and at-risk populations. In terms of study design, we included all types of empirical studies (cross-sectional, longitudinal, interventional, validation studies). Qualitative studies and case reports were excluded.

### Information Sources and Search Strategy

We conducted the main search and selection process between May 2021 and October 2021, identifying original studies by searching the electronic databases APA PsycInfo (Ovid) and MEDLINE (Ovid). On February 25, 2022, an updated search for papers published after the initial search was conducted and resulted in the addition of nine reports. The references of all selected publications were searched for additional studies. We included additional sources on psychometric data in our assessment of psychometric quality if it was referred to in one of the publications and available in English. Table 1 presents the search strategy used via the Ovid database. A librarian was consulted to develop and improve the search strategy.

**Table 1:** Search Strategy (5 May 2021; Databases: Ovid Medline(R), Ovid APA Psycinfo)

1	(Parent–child* or parent-adolescent* or parent-teen* or mother–child* or father-child* or mother-adolescent* or father-adolescent* or caregiver-child* or caregiver-adolescent*).ti,ab,hw,kf,mh
2	Communication.ti,ab,hw,kf,mh
3	(Functioning or well-being or mental health or stress or psychopathol* or adjust* or relationship or internali* or externali* or valid* or psychometr* propert*).ti,ab,hw,kf,mh
4	1 and 2 and 3
5	4 and 1991:2022.(sa_year)
6	Remove duplicates from 5

### Selection Process

Bibliographical data were uploaded to Rayyan (rayyan.ai) for masked screening. Pairs of team members (HZ, KF, JB, SC, SO, YH) screened titles and abstracts. Full-texts retrieved after screening were checked for eligibility by the same pairs independently, again using Rayyan. Disagreements were resolved by discussion.

### Data Collection Process and Data Items

Data items extracted from the studies were scale name, sample description, child sample age, sampling strategy, the focus of the paper, main method, relation of parent–child communication to other constructs, and main results about parent–child communication. To conduct the quality assessments, reported psychometric properties were also extracted (see results section). Multiple reports from a study/sample were treated as a single study. Data were extracted by KF, SO, SC, YH, and JB. All extracted data were completely cross-checked by HZ.

### Quality Assessment

In our evaluation of instrument quality, we relied on the criteria set forward by Hunsley and Mash (2007, 2008, 2018), complemented by Youngstrom et al. (2017), and summarized in De los Reyes and Langer (2018). This system is used to rate the psychometric properties of assessment instruments across nine categories: (a) norms, (b) internal consistency, (c) interrater reliability, (d) test–retest reliability, (e) content validity, (f) construct validity, (g) validity generalization, (h) treatment sensitivity, and (i) clinical utility. Each category includes a description of the quality of evidence required for a rating of adequate (minimal level of scientific rigor), good (solid scientific support), or excellent (extensive, high-quality support). Youngstrom et al. (2017) later added repeatability, discriminative validity, and prescriptive validity to the original system. Since the original system was intended for clinical measures, not all categories apply to parent–child communication scales. Thus, in the current review, we rated the following nine quality categories: norms, internal consistency, test–retest reliability, content validity, construct validity, factorial structure, discriminative validity, validity generalization, and treatment sensitivity (See Supplement Table).

In terms of norms, there are no clear cut-offs for sample size, but we considered community samples *N* > 400 and clinical samples *N* > 100 as representative. In terms of reliability, the system applies the following criteria for Chronbach’s α: 0.70—0.79 is adequate, 0.80 to 0.89 is good, and > 0.90 is excellent, based on the median of reported numbers. The quality assessment was conducted by KF, SO, SC, YH, and JB, as well as cross-checked by SC and HZ. If members of the review team had co-authored a paper under consideration, the other team members did the quality assessment.

## Results

### Study Selection

**Fig. 1:**
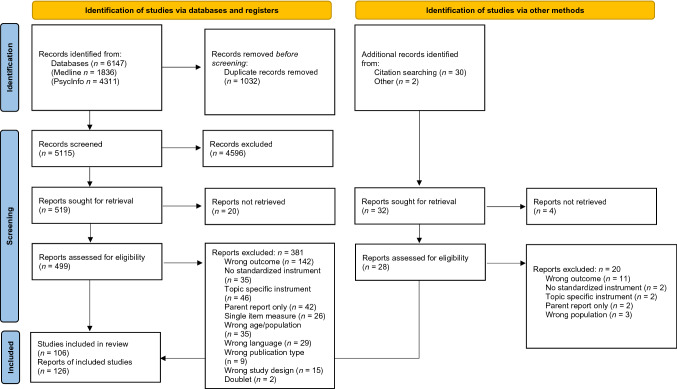
PRISMA flow diagram according to Page et al. (2021)

A total of 6147 hits were retrieved from the databases, and 1032 duplicates were removed. Based on the screening of the remaining 5115 titles and abstracts, 499 full-texts were retrieved. Based on these we included 118 papers. In addition, reference lists were searched for eligible literature, resulting in 32 papers of which 28 were retrieved. Eight additional papers were included, resulting in 126 papers in total. Figure 1 shows the corresponding PRISMA flow chart. Table 2 provides an overview of included articles.

**Table 2:** Overview of included parent–child communication studies

Paper		Scale	Country	*N*	% fem	Sample description	Sample age in years			Parent–child communication *M* (*SD*)
Author	Year						Range	M	SD	
Acuña and Kataoka	(2017)	PACS	USA	98	44.9	Community	n.a	13.1	1.2	Open 36.1 (9.9); problem 28.8 (8.4)
Asfour et al.	(2017)	PACS	USA	959	45.3	Community^a^	n.a	13.8	0.7	68.9 (14.7)
Bartlett et al.	(2016)	PACS	USA	101	100	Community	11–15	12.6	0.9	69.6 (13.2)
Bireda and Pillay	(2018)	PACS	Ethiopia	809	47.1	Community	n.a	16.8	1.6	*Not interpretable
Brage et al.	(1993)	PACS	USA	156	60.3	Community	11–18	14.0	1.6	
Cai et al.	(2021)	PACS	China	1439	45.7	Community^b^	n.a	12.4	1.2	
Caprara et al.	(2005)	PACS	Italy	380	51.3	Community	n.a	15.0^‡^	n.a	*Not interpretable
Carbonero et al.	(2017)	PACS	Spain	271	46.9	Community	11–15	n.a	n.a	
Chen et al.	(2019)	PACS	China	1134	46.8	Community	11–16	13.2	1.1	*Not interpretable
Cho et al.	(2018)	PACS	South Korea	944	50.4	Community	16–18	16.5	0.6	*Not interpretable
Clark and Shields	(1997)	PACS	USA	339	53.1	Community	14–19	16.2	n.a	Open 29.6–40.1; problem 30.4–37.9
Collin et al.	(1995)	PACS	USA	78	53.8	Community	12–18	15.6	0.7	parent 66.1 (13.5); stepparent 57.7 (15.0)
Cordova et al.	(2014)	PACS	USA	746	47.9	Community	n.a	13.9	n.a	69.9 (14.8)
Cornell et al.	(1991)	PACS	USA	44	100	Community	13–17	14.9	n.a	Mother 68.9 (17.8); father 64.7 (19.5)
Daley	(2006)	PACS	USA	40	n.a	Clinical & community	n.a	13.7^§^	2.3^§^	Several means reported
De Los Reyes et al.	(2016)	PACS	USA	141	57	Community^c^	15–18	17.0	0.7	Several means reported
Dickerson and Crase	(2005)	PACS	USA	18	44.4	Clinical	14–17	n.a	n.a	*Not interpretable
Estévez et al.	(2005)	PACS	Spain	983	52.8	Community^d^	11–16	13.7	n.a	
Farrell and Barnes	(1993)	PACS	USA	699	n.a	Community	13–16	n.a	n.a	Open 27.9–47.3; problem 20.7–33.0
Finan et al.	(2018)	PACS	USA	372	55	Community	n.a	16.1^‡^	0.7	62.0 (17.2)–67.0 (17.2)
Gosselin and David	(2007)	PACS	Canada	80	68.8	Community	n.a	14.0	2.0	Boys 69.2 (12.3), girls 66.7 (13.1)
Hadley et al.	(2013)	PACS	USA	71	n.a	Clinical	13–18	14.9	1.3	Open 35.5 (11.2); problem 29.8 (8.4)
Hartos and Power	(1997)	PACS	USA	161	50.3	Community	14–15	n.a	n.a	64.1 (13.0); subscales also reported
Hartos and Power	(2000a)	PACS	USA	161	50.3	Community^e^	14–15	n.a	n.a	
Hartos and Power	(2000b)	PACS	USA	82	43	Community	13–15	13.9	0.8	63.4 (12.8)
Heller et al.	(2006)	PACS	USA	236	59	Community	14–16	14.8	0.7	Open (mother) 33.8 (9.25); (father) 37.9 (8.6)
Henry and Lovelace	(1995)	PACS	USA	95	58.9	Community	14–18	16.1	1.2	68.9 (16.2)
Herrero et al.	(2006)	PACS	Spain	973	52.8	Community^f^	11–16	13.7	1.6	Several means reported
Hill and Roberts	(2019)	PACS	USA	167	44.3	Community	11–19	n.a	n.a	*Not interpretable
Houck et al.	(2007)	PACS	USA	38	65.8	At risk	12–17	14.9	1.7	36.2 (1.5)-55.0 (10.5)
Howard et al.	(1999)	PACS	USA	333	43	Community	9–15	n.a	n.a	*not interpretable
Howard Sharp et al.	(2020)	PACS	USA	132	56.8	At risk & community^g^	10–18^§^	13.2^§^	2.2^§^	Several means reported
Huizinga et al.	(2005)	PACS	Netherlands	212	56.6	At risk	11–18	15.1	2.3	Open 39.7 (6.6)–37.4 (7.2); problem 36.5 (7.4)–35.4 (5.8)
Jackson et al. (Study I)	(1998)	PACS	Netherlands	413	56.2	Community	13–17	n.a	n.a	Mother 77.6 (10.5); father 72.5 (11.2); subscales also reported
Jackson et al. (Study II)	(1998)	PACS	Netherlands	660	53.3	Community	13–15	n.a	n.a	Mother 74.8 (11.9); father 71.7 (12.8); subscales also reported
Jeong and Chun	(2010)	PACS	South Korea	578	55.5	Community	n.a	16.2	n.a	
Jiménez et al.	(2019)	PACS	Spain	2399	50	Community	11–20	14.7	1.8	*Not interpretable
Keim et al.	(2017)	PACS	USA	185	52.4	At risk & community^h^	10–17	13.5	2.4	Open 43.7(7.1)–33.9(11.0); problem 27.8(9.3)–22.9(7.3)
Kim and Park	(2011)	PACS	USA	77	39	Community	11–15	12.9	1.1	
Kim et al.	(2018)	PACS	South Korea	402	44.5	Community	n.a.‖	n.a	n.a	Mother 63.0 (13.2); father 69.8 (15.1)
Kimiecik and Horn	(2012)	PACS	USA	173	58	Community	9–12	10.0	0.7	*Not interpretable
Knight et al.	(1992)	PACS	USA	303	n.a	Community	8–14	10.5	1.1	
Lambert and Cashwell	(2004)	PACS	USA	100	58	Community	10–13	n.a	n.a	Mother 76.3 (14.6); father 68.2 (17.3)
Liu et al.	(2019)	PACS	China	2751	50.5	Community	13–19	14.9	1.9	*Not interpretable
López et al.	(2006)	PACS	Spain	843	53	Community^i^	11–16	13.7	n.a	*Not interpretable
Lu et al.	(2020)	PACS	China	464	45.7	Community^j^	11–17	n.a	n.a	Several means reported
Malcolm et al.	(2013)	PACS	USA	171	26.9	Community^k^	n.a	14.9	1.2	63.7 (13.4)
Manczak et al.	(2018)	PACS	USA	194	100	Clinical	12–16	14.5	1.2	
Marta	(1997)	PACS	Italy	279	53.8	Community	16–19	17.0	n.a	Several means reported
McNaughton et al.	(2015)	PACS	USA	53	49.1	Community	9–14	11.0	n.a	Mother 68.3 (7.9)–69.5 ( 9.5)
de la Rubia and Morales	(2012)	PACS	Mexico	198	43.4	Community	14–17	15.0	1.4	54.0 (10.8)–63.7 (11.4); Subscales also reported
Morrison and Zetlin	(1992)	PACS	USA	60	50	At risk & community	15–18	n.a	n.a	50.9 (11.8)–83.3 (5.0)
Noor and Alwi	(2013)	PACS	Malaysia	197	61.9	Community	12–16	13.8	1.5	Mother 68.7 (11.1)
Ochoa et al.	(2007)	PACS	Spain	1068	53	Community	11–16	13.7	1.6	
Ochoa et al.	(2021)	PACS	USA	456	52.9	Community^l^	12–16	13.9	1.4	76.3 (10.1)
Ohannessian and Vannucci	(2020)	PACS	USA	1057	53	Community	n.a	16.2^‡^	0.8	Mother 67.1 (15.3); father 64.0 (16.2)
Ohannessian	(2012)	PACS	USA	683	57	Community^m^	15–17^‡^	16.1^‡^	0.7	Mother 66.8 (13.9); father 65.3 (15.5)
Otero et al.	(2011)	PACS	Spain	198	n.a	(1)Clinical & (2)community	9–17	(1)15.5; (2)15.2	(1)1.7; (2)1.9	67.6 (13.2)–82.2 (9.7); subscales also reported
Pantaleao and Ohannessian	(2019)	PACS	USA	980	53	Community^n^	n.a	16.2^‡^	0.8	Mother 66.8 (14.1); father 64.4 (15.5)
Park and Kim	(2012)	PACS	USA	166	45.8	Community	11–15	13.0	1.2	Mother 69.0 (13.4); father 64.9 (13.0)
Phillips-Salimi et al	(2014)	PACS	USA	70	38.6	At risk	11–19	14.8	1.7	71.6 (11.7)
Prado et al.	(2013)	PACS	USA	213	36.2	Community	n.a	13.8	0.8	
Qu et al.	(2021)	PACS	China	842	47.9	Community	n.a	12.2	0.5	*Not interpretable
Reis and Heppner	(1993)	PACS	USA	31	100	Clinical & community	n.a	15.0	1.6	Clinical 57.9 (18.5); community 70.4 (18.5)
Rhee et al.	(2003)	PACS	USA	189	55.6	Community	13–18	15.5	1.2	
Rishel et al.	(2010)	PACS	USA	518	70	Community	12–17	15.0	1.3	*Not interpretable
Ritchwood et al.	(2017)	PACS	USA	465	55.5	Community	10–14	12.6	1.4	*Not interpretable
Rosenthal et al.	(2001)	PACS	n.a	n.a	n.a	Community	8th–10th grade	n.a	n.a	*Not interpretable
Rosnati et al.	(2007)	PACS	Italy	276	51.4	Community	11–17	13.8	n.a	Mother 73.7 (13.3); father 71.9 (13.9)
Scabini et al.	(1999)	PACS	Italy	692	51.8	Community	n.a	n.a	n.a	56.0 (13.1)–79.3 (10.1)
Schuster et al.	(2013)	PACS	USA	1145	57.7	Community	15–19	16.9	n.a	*Not interpretable
Schwinn et al.	(2014)	PACS	USA	67	100	Community	10–12	11.9	0.9	*Not interpretable
Sears et al.	(2020)	PACS	Canada	259	52.9	Community	12–14	13.0	0.7	65.0 (11.3) -73.4 (11.4)
Shin et al.	(2010)	PACS	South Korea	178	52.8	Community	10–13	13.0	0.4	*Not interpretable
Simpson et al.	(2020)	PACS	USA	1034	55	Community^o^	n.a	16.2	0.8	Mother 67.3 (16.3); father 63.7 (16.3)
Toombs et al.	(2018)	PACS	Canada	18	72	Community	13–17	n.a	n.a	
Vannucci et al.	(2019)	PACS	USA	100	40	Community	13–17	15.1	1.0	
Varela et al.	(2009)	PACS	USA, Mexico	217	n.a	Community	9–14	11.3	5.5	Open 35.8 (10.6)–41.1 (7.6)
Velazquez et al.	(2021)	PACS	USA	101	52	Community	n.a	14.4	1.9	54.0 (7.7)
Wang et al.	(2013)	PACS	Bahamas	913	n.a	Community	10–14	10.4	n.a	*Not interpretable
Wang et al.	(2019)	PACS	China	1969	45.6	Community^p^	11–17	13–13.2	n.a	54.1 (9.2)–57.3 (10.1)
Wang et al.	(2020)	PACS	China	4565	43.4	Community	n.a	13.0	1.3	61.0 (11.2)–54.1 (9.2); subscales also reported
Weber et al.	(2019)	PACS	Sweden	23	65.2	At risk	12–20	16.2	1.9	63.4 (6.5)
White and Matawie	(2004)	PACS	Australia	218	72.5	Community^q^	14–19	16.9	1.2	Mother 67.5 (14.5); father 63.1 (15.1)
White	(2000)	PACS	Australia	271	64.2	Community	14–19	16.8	2.8	
Wu and Chao	(2011)	PACS	USA	634	48.9	Community	14–18	16.0	0.6	
Xia et al.	(2004)	PACS	China	768	64	Community	12–19	16.2	2.5	
Xiao et al.	(2011)	PACS	USA	336	42.6	Community	9–15	n.a	n.a	
Yang et al.	(2007)	PACS	USA	817	56.5	Community	13–16	n.a	n.a	*Not interpretable
Yoon	(2000)	PACS	USA	241	n.a	Community	12–19	14.0	n.a	
Young and Childs	(1994)	PACS	USA	171	73	(1)Clinical & (2)community	(1)14–18; (2)14–19	(1)16.1; (2)16.4	n.a	Clinical: mother 55.4 (15.5); father 51.9 (15.5)
Yu et al.	(2006)	PACS	Bahamas	752	54	Community^r^	n.a	10.5	n.a	
Zhou et al.	(2021)	PACS	China	620	60.2	Community	12–19	15.6	1.6	Open 31.2 (8.1); problem 25.6 (6.7)
Bandura et al.	(2011)	PACS†	Italy	142	45.1	Community	13–19	16.0	n.a	*Not interpretable
Berzonsky et al.	(2007)	PACS†	Netherlands	281	54.8	Community	n.a	13.3	0.5	
Branje	(2008)	PACS†	Netherlands	30	100	Community	12–13	n.a	n.a	*Not interpretable
Ioffe et al.	(2020)	PACS†	USA	400	54	Community	11–14	12.5	1.0	Open: mother 38.5 (9.9); father 34.3 (9.8)
Lin et al.	(2021)	PACS†	China	1539	54.2	Community	n.a	15.8	0.5	*Not interpretable
Rodrigues et al.	(2020)	PACS†	USA	95	100	Community	13–18	15.4	0.1	*Not interpretable
Updegraff et al.	(2002)	PACS†	USA	143	52	Community	9–12	10.7	0.7	*Not interpretable
Van Dijk et al.	(2014)	PACS†	Netherlands	323	51.1	Community	12–15	13.3	0.5	*Not interpretable
Portugal and Alberto	(2019)	COMPA	Portugal	72	43.06	(1)At risk & (2)community	(1)12–16; (2)7–16	(1)11.4; (2)10.9	(1)2.8; (2)2.1	
Kwok and Shek	(2010a)	FACS/ MACS	Hongkong	5557	46.9	Community	11–18	13.9	1.5	
Kwok and Shek	(2010b)	FACS/ MACS	Hongkong	5557	46.9	Community^s^	n.a	n.a	n.a	Mother 69.3 (13.9); father 60.5 (13.5)
Kwok and Shek	(2011)	FACS/ MACS	Hongkong	5557	46.9	Community^t^	n.a	n.a	n.a	
Yang et al.	(2013)	FCP	USA	163	53.4	Community	15–17	n.a	n.a	
Carson et al.	(1999)	PACI (Bienvenu)	India	107	46.73	Community	12–16	13.7	0.7	83.5 (12.6)
Green and Vosler	(1992)	PACI (Bienvenu)	USA	39	28.2	At risk	12–19	15.4	1.5	80.4–84.4
Hill and Roberts	(2019)	PACI (Bienvenu)	USA	167	44.31	Community	11–19	n.a	n.a	
Raimundi et al.	(2019)	PACI (Schmidt)	Argentina	476	n.a	Community	11–19	15.2	1.5	Open 23.3(6.2)–31.6(3.1); problems 11.5 (3.7)–20.9(6.1); restricted 18.5(5.6)–27.5(4.5)
Ying et al.	(2015)	PCCQ	China	3349	48.6	Community	12–15	n.a	n.a	
Ying et al.	(2018)	PCCQ	China	437	45	Community	n.a	10.9	0.7	*Not interpretable
Ying et al.	(2019)	PCCQ	China	437	45	Community^u^	n.a	10.9	0.7	*Not interpretable
Zhang et al.	(2019)	PCCS (Chi)	China	296	54.7	Community	n.a	13.1	1.1	
Blanc et al.	(2021)	PCCS (Krohn)	Spain	360	58.7	Community	12–19	15.2	1.4	*Not interpretable
Davidson and Cardemil	(2009)	PCCS (Krohn)	USA	40	52.5	Community	10–14	12.2	1.6	22.0 (4.0)
Fite et al.	(2014)	PCCS (Loeber)	USA	289	0	Community	n.a	16.0	n.a	48.1 (8.6)
Schulte et al.	(2017)	PCCS (Loeber)	USA	102	50	At risk	n.a	9.7	2.5	36.9 (7.4)
Fjermestad et al.	(2020)	PCCS (McCarty)	Cambodia	52	44	At risk	8–21	12.7	2.7	
Offrey abd Rinaldi	(2017)	PCCS (McCarty)	Canada	225	60	Community	n.a	12.7	n.a	
Orm et al.	(2021)	PCCS (McCarty)	Norway	123	52	At risk^v^ & community^w^	8–16	11.0–11.5	2.0–2.5	27.9 (6.2)–34.3 (4.7)
Orm et al.	(2022)	PCCS (McCarty)	Norway	145	49.7	At risk^x^ & community	8–16	11.5	2.2	Child communication 10.2 (3.1)–13.1 (2.2); parent communication 18.8 (3.6)–21.4 (2.8)
Fredriksen et al.	(2021)	PCCS† (McCarty)	Norway	107	54.6	At risk	8–16	11.5	2.1	
Haukeland et al.	(2021)	PCCS† (McCarty)	Norway	100	50	At risk^y^ & community	8–16	11.5	2.2	*Not interpretable
Haukeland et al.	(2020)	PCCS† (McCarty)	Norway	99	54.5	At risk^z^	8–16	11.5	2.0	
Fitzpatrick and Ritchie	(1994)	RFCP	USA	168	n.a	Community	7th–11th grade	n.a	n.a	*Not interpretable
O'Toole et al.	(2021)	RFCP	Ireland	47	n.a	At risk	8–18	13.2	2.8	
Sillars et al.	(2005)	RFCP	USA	50	44	Community	11–14	n.a	n.a	

*fem*. Female, *PCC*  parent–child communication

^†^Only subscale used

^‡^Value reported at T1

^§^Same values for both subsamples. ‖First year middle school, not otherwise specified

*Not interpretable: means are provided but not comparable due to changes in scale or calculation

^a^Comprises samples of Cordova et al. (2014) and Prado et al. (2013)

^b^Subsample of Wang et al. (2020)

^c^Subsample of Finan et al. (2018)

^d^Subsample of Ochoa et al. (2007)

^e^Same sample as Hartos and Power (1997)

^f^Subsample of Ochoa et al. (2007)

^g^Same community sample as in Keim et al. (2017)

^h^Same community sample as in Howard Sharp et al. (2020)

^i^Subsample of Ochoa et al. (2007)

^j^Subsample of Wang et al. (2020)

^k^Subsample of Prado et al. (2013)

^l^Subsample of Cordova et al. (2014)

^m^Subsample of Ohanessian and Vanucci (2020)

^n^Subsample of Ohanessian and Vanucci (2020)

^o^Subsample of Ohanessian and Vanucci (2020)

^p^Subsample of Wang et al. (2020)

^q^Subsample of White (2000)

^r^Subsample of Wang et al. (2013)

^s^Same as Kwok (2010a)

^t^Same as Kwok (2010a)

^u^Same sample as Ying et al. (2018)

^v^Subsample of Fredriksen et al. (2021)

^w^Subsample of Orm et al. (2022)

^x^Subsample of Fredriksen et al. (2021)

^y^Subsample of Fredriksen et al. (2021)

^z^Subsample of Fredriksen et al. (2021)

### Study Characteristics and Identified Instruments

Twelve different instruments were identified (see Table 3 for an overview and Table 4 for quality assessment). The Parent-Adolescent Communication Scale (PACS; Barnes & Olson, 1982) was used in 100 papers based on 85 studies. The Parent–Child Communication Scale (PCCS; McCarty, McMahon and Conduct Problems Prevention Research Group, 2003) was used in seven papers based on four studies. The Parent-Adolescent Communication Inventory (PACI; Bienvenu, 1969) and the Revised Family Communication Patterns Instrument (RFCP; Ritchie & Fitzpatrick, 1990) were used in three studies each. The Parent–Child Communication Questionnaire (PCCQ; Yang & Zou, 2008), the Parent–Child Communication Scale (PCCS; Loeber et al., 1998; also known under the name Revised Parent-Adolescent Communication Form (RPACF); Loeber et al., 1991), and the Parent–Child Communication Scale (PCCS; Krohn et al., 1992) were all used in two studies each. The Father-Adolescent/Mother-Adolescent Communication Scale (FACS/MACS; Shek et al., 2006), the Parent–Child Communication Scale (PCCS; Chi, 2011), the Perception of Parenting Communication Scale (COMPA; Portugal & Alberto, 2014), the Parent-Adolescent Communication Inventory (PACI; Schmidt et al., 2010), and the Family Communication Patterns Scale (FCP; McLeod et al., 1972) were all used in one study each.

**Table 3:** Overview of parent–child communication measures

Scale name	Abbreviation	Language	Original reference	# items	Subscales	*n* studies	*n* papers
Perception of Parenting Communication Scale	COMPA	Portuguese	Portugal and Alberto (2014)	16 (child), 39 (adoelscent), 44 (parent version)	Parental availability to communication, children confidence/sharing, emotional support/affective expression, meta-communication, negative communication patterns	1	1
Father-Adolescent Communication Scale/Mother-Adolescent Communication Scale	FACS/MACS	Chinese	Shek et al. (2006)	25	n.a	1	3
Family Communication Pattern Scale	FCP	English	McLeod et al. (1972)	10	Socio-orientation, concept-orientation	1	1
Parent-Adolescent Communication Inventory	PACI (Bienvenu)	English	Bienvenu (1969)	40	n.a	3	3
Parent-Adolescent Communication Inventory	PACI (Schmidt)	Spanish	Schmidt et al. (2010)	42	Open, problem, restricted	1	1
Parent-Adolescent Communication Scale	PACS	English	Barnes and Olson (1982)	20	Open, problem	51	55
Parent-Adolescent Communication Scale	PACS	Chinese		20	Open, problem	4	7
Parent-Adolescent Communication Scale	PACS	Dutch		20	Open, problem	6	5
Parent-Adolescent Communication Scale	PACS	French		20	Open, problem	1	1
Parent-Adolescent Communication Scale	PACS	Italian		20	Open, problem	5	5
Parent-Adolescent Communication Scale	PACS	Khmer		20	Open, problem	1	1
Parent-Adolescent Communication Scale	PACS	Korean		20	Open, problem	4	4
Parent-Adolescent Communication Scale	PACS	Malay		20	Open, problem	1	1
Parent-Adolescent Communication Scale	PACS	Spanish		20	Open, offensive, avoidant	5	8
Parent-Adolescent Communication Scale	PACS	Swedish		20	Open, problem	1	1
Parent‐Child Communication Questionnaire	PCCQ	Chinese	Yang and Zou (2008)	19 or 23	Open expression, listening to parents, conflict resolution, mutual understanding	2	3
Parent–Child Communication Scale (Chi)	PCCS (Chi)	Chinese	Chi (2011)	12	Relationship-oriented communication, problem-solving oriented communication	1	1
Parent–Child Communication Scale (Krohn)	PCCS (Krohn)	English	Krohn et al. (1992)	7	n.a	1	1
Parent–Child Communication Scale (Krohn)	PCCS (Krohn)	Spanish		7	n.a	1	1
Parent–Child Communication Scale (Loeber)	PCCS (Loeber)	English	Loeber et al. (1998)	29	n.a	2	2
Parent–Child Communication Scale (McCarty)	PCCS (McCarty)	English	McCarty et al. (2003)	10 (child), 20 (parent version)	Child communication, parent communication	1	1
Parent–Child Communication Scale (McCarty)	PCCS (McCarty)	Khmer		10 (child), 20 (parent version)	Child communication, parent communication		
Parent–Child Communication Scale (McCarty)	PCCS (McCarty)	Norwegian		8	Child communication, parent communication	2	5
Revised Family Communication Patterns Instrument	RFCP	English	Ritchie and Fitzpatrick (1990)	26	Conversation, conformity	3	3

**Table 4:** Psychometric quality assessment of parent–child communication measures

Scale name	Abbr	Language	# studies	Norms	α	Test–retest rel	Cont. val	Cons. val	FA	Discr. val	Val. gen	T.- sen	Σ
Perception of Parenting Communication Scale	COMPA	Portuguese	1	n.a	0	n.a	1	0	n.a	n.a	n.a	n.a	1
Father–Mother–Adolescent Communication Scale	FACS/MACS	Chinese	1	1	3	n.a	n.a	0	n.a	n.a	n.a	n.a	4
Family Communication Pattern Scale	FCP	English	1	n.a	1	n.a	n.a	n.a	n.a	n.a	n.a	n.a	1
Parent–Adolescent Communication Inventory	PACI (Bienvenu)	English	3	n.a	0	1	0	n.a	n.a	n.a	1	0	2
Parent–Adolescent Communication Inventory	PACI (Schmidt)	Spanish	1	n.a	2	n.a	0	n.a	0	n.a	0	n.a	2
Parent–Adolescent Communication Scale	PACS	English	51	2	2	1	1	0	1	0	3	1	11
Parent–Adolescent Communication Scale	PACS	Chinese	4	1	3	n.a	1	n.a	1	n.a	2	n.a	8
Parent–Adolescent Communication Scale	PACS	Dutch	6	1	1	n.a	1	n.a	1	n.a	2	n.a	6
Parent–Adolescent Communication Scale	PACS	French	1	n.a	n.a	n.a	1	n.a	n.a	n.a	n.a	n.a	1
Parent–Adolescent Communication Scale	PACS	Italian	5	1	1	n.a	1	n.a	n.a	n.a	1	n.a	4
Parent–Adolescent Communication Scale	PACS	Khmer	1	n.a	1	n.a	1	n.a	n.a	n.a	n.a	n.a	2
Parent–Adolescent Communication Scale	PACS	Korean	4	1	2	n.a	1	n.a	n.a	n.a	1	n.a	5
Parent–Adolescent Communication Scale	PACS	Malay	1	n.a	2	n.a	1	n.a	n.a	n.a	n.a	n.a	3
Parent–Adolescent Communication Scale	PACS	Spanish	5	1	2	n.a	1	n.a	1	n.a	1	n.a	6
Parent–Adolescent Communication Scale	PACS	Swedish	1	n.a	2	n.a	1	n.a	n.a	n.a	n.a	n.a	3
Parent–Child Communication Questionnaire	PCCQ	Chinese	2	n.a	1	n.a	n.a	1	n.a	n.a	1	n.a	3
Parent–Child Communication Scale (Chi)	PCCS (Chi)	Chinese	1	0	2	n.a	n.a	0	n.a	n.a	n.a	n.a	3
Parent–Child Communication Scale (Krohn)	PCCS (Krohn)	English	1	n.a	1	n.a	n.a	n.a	n.a	n.a	n.a	n.a	1
Parent–Child Communication Scale (Krohn)	PCCS (Krohn)	Spanish	1	n.a	2	n.a	n.a	n.a	n.a	n.a	n.a	n.a	2
Parent–Child Communication Scale (Loeber)	PCCS (Loeber)	English	2	n.a	1	n.a	n.a	n.a	n.a	n.a	n.a	n.a	1
Parent–Child Communication Scale (McCarty)	PCCS (McCarty)	English	1	1	0	0	1	n.a	n.a	0	1	0	3
Parent–Child Communication Scale (McCarty)	PCCS (McCarty)	Khmer	1	n.a	0	n.a	1	n.a	n.a	n.a	n.a	n.a	1
Parent–Child Communication Scale (McCarty)	PCCS (McCarty)	Norwegian	2	0	1	n.a	1	n.a	0	n.a	1	1	4
Revised Family Communication Patterns Instrument	RFCP	English	3	n.a	2	1	0	1	n.a	n.a	1	n.a	4

# number, α internal consistency, *Rel.* reliability, *Cont.* content, *Val*. Validity, *Cons*. Construct, *FA* factor structure, *Discr*. Discriminant, *Gen*. generalization, *T-sen*. treatment- sensitivity, Σ sum

#### The Parent–Adolescent Communication Scale (PACS)

The PACS (Barnes & Olson, 1982) was the most widely used instrument and has been translated from English into nine other languages (Spanish, Dutch, Chinese, French, Malay, Italian, Khmer, Korean, and Swedish). The instrument comprises two subscales (open communication and communication problems, 10 items each). Items are measured on a 5-point Likert scale, and parent and children versions are identical apart from changing referents (my mother/father/daughter/son). For the quality assessment, Barnes and Olson's (1982) study was considered as source in addition to the studies retrieved by the systematic literature search. In terms of content validity, the original authors specified the conceptual foundation within the framework of the circumplex model of family functioning: parent–child communication was conceptualized as an additional dimension facilitating adaptive change in family functioning (Barnes & Olson, 1985). However, in most studies found in this review, the PACS was used as a stand-alone measure of dyadic parent–child communication. The factorial structure found in the initial study (Barnes & Olson, 1982) was corroborated by a principal component analysis in a Dutch study (Jackson et al., 1998). In a Spanish sample, however, three factors were found in another principle component analysis (open communication, communication problems, and avoidant communication; Estevez et al., 2005). In an American sample, only one factor was found in exploratory and confirmatory factor analyses, with two items from the problem scale not loading on the same factor as the other items (Wu & Chao, 2011). The PACS has shown good internal consistency in most of its’ versions. The means and standard deviations are available for large samples (i.e., *n* > 400) in six versions (see Table 2). For the English version of the PACS, some evidence on test–retest reliability and treatment sensitivity was found. The included studies did not report sufficiently on construct and discriminative validity. The PACS has been published originally in Barnes and Olson (1982) and is available online (for example: https://scales.arabpsychology.com/s/parent-adolescent-communication-scale-pacs/).

#### The Parent–Child Communication Scale (PCCS McCarty)

The PCCS (McCarty, McMahon and Conduct Problems Prevention Research Group, 2003) was the second most widely used instrument. It has been translated from English into Norwegian and Khmer. Parent and child versions are different; the child version consists of 10 items (parents 20 items) in the original version and is based on a 5-point Likert scale. For quality assessment, Pek (2006) was considered as additional source. With regard to content validity, the instrument is intended to assess the parent’s openness to communication (Pek, 2006), but the conceptual foundation is not specified further. In later analyses, only eight items fit the subscales parent and child communication (five and three items, respectively). This factorial structure was corroborated by confirmatory factor analyses in a Norwegian study (Orm et al., 2022). Internal consistency is between low and adequate. For the English version, norm values of a large sample exist (Pek, 2006), means and standard deviations for an at-risk sample can be found in Orm et al. (2021, 2022). There was no sufficient evidence with regard to test–retest reliability, construct, and discriminative validity, but for treatment sensitivity. The PCCS is available from the website of the Fast Track Project (https://fasttrackproject.org/data-instruments). The questionnaire is not included in the original reference publication (McCarty et al., 2003).

#### The Parent–Adolescent Communication Inventory (PACI Bienvenu)

The PACI (Bienvenu, 1969) is explicitly recommended for children older than 13 years. According to Carson et al. (1999) and Green and Vosler (1992), it consists of 40 items, whereas the original author claims it to be a 36-item scale. No subscales are hypothesized nor has a factorial structure been reported. The original article (Bienvenu, 1969) was considered as an additional source for quality assessment. Psychometric data on test–retest reliability have been presented (Bienvenu, 1969). Green and Vosler (1992) reported treatment sensitivity with regard to the parent, but not the child scale. Internal consistency is less than adequate. In the present sample of studies, there was no information on content, discriminative, and construct validity for the PACI. A 21-item version of the scale is available in the original publication (Bienvenu, 1969).

### The Revised Family Communication Patterns Instrument (RFCP)

The RFCP (Ritchie & Fitzpatrick, 1990) consists of 26 items and has two subscales: conformity and conversation orientation (11 and 15 items). For the quality assessment, Ritchie and Fitzpatrick's (1990) study was considered as an additional source. The factorial structure has not been explored and there is no information on treatment sensitivity. Internal consistency is good, and test–retest reliability is adequate. In the present sample of studies, there was no information on content, discriminative, and construct validity for the RFCP. The RFCP is available online (http://dx.doi.org/10.13140/RG.2.2.15136.64000) and also included in the original publication (Ritchie & Fitzpatrick, 1990).

#### Further Instruments

With regard to the remaining instruments, the PCCS (Chi, 2011) and the PCCQ consist of two and four subscales, respectively. The COMPA is recommended for ages seven to 18 and has five subscales: Parental availability to communication, children confidence/sharing, emotional support/affective expression, meta-communication, and negative communication patterns (Portugal & Alberto, 2014). The factor structures have not been established for the PCCS (Chi), the PCCQ, or the COMPA. The means and standard deviations in larger samples (i.e., *n* > 400) were only reported for the FACS/MACS (Shek et al., 2006) and for the PCCS (Chi, 2011). The internal consistencies ranged from less than adequate (COMPA; Portugal & Alberto, 2014) over adequate (FCP; McLeod et al., 1972; PCCQ; & Zou, 2008; PCCS; Krohn et al., 1992; PCCS; Loeber et al., 1998) and good (PACI; Schmidt et al., 2010; PCCS; Chi, 2011) to excellent (FACS/MACS; Shek et al., 2006). Test–retest reliability, content validity, construct validity, factorial structure, discriminative validity, and treatment sensitivity were not reported in any of the included studies. To our knowledge, complete original items are only available for COMPA and PCCS (Krohn) in Portugal and Alberto (2014) and Krohn et al. (1992), respectively.

### Synthesis: Psychometric Evidence of Parent–Child Communication Measures

We extracted the available psychometric data from all publications. We included additional sources on psychometric data in our assessment of the psychometric quality for four measures (i.e., PACS, PCCS (McCarty), PACI, (Bienvenu), RFCP; i.e., Barnes & Olson, 1982, 1985; Bienvenu, 1969; Ritchie & Fitzpatrick, 1990; Pek, 2006). Most instruments showed at least adequate internal consistency. Means and standard deviations that can be considered as normative information (community samples *N* > 400 and clinical samples *N* > 100) were reported for almost half of the existing measures (Table 4). The conceptual and theoretical background of the instruments were often not specified clearly. Additional psychometric information is scarce, and construct and discriminative validity were not stated in an explicit way for any of the instruments. However, means (Table 2) indicate that PACS and PCCS (McCarty) can detect differences in the quality of parent–child communication between community, at-risk, and clinical samples.

## Discussion

The aim of this review was to identify instruments that measure parent-child communication from the child’s perspective as well as which samples they have been applied to and to assess the psychometric quality of these instruments. We identified twelve instruments across 106 studies. The PACS had been used in most studies (*k* = 85) by far. This means there are relatively few multiple-item and general (i.e., not topic-specific) child-rated parent–child communication instruments, given that we included > 100 studies in this review. On the one hand, this indicates some unification in the field, with a strong PACS dominance. Given that the other instruments were used in four studies each or less, with five instruments having only been used in single studies, a potential conclusion could be that the PACS should be considered the “gold standard” for child-reported parent–child communication quality. However, frequency does not ensure quality, which will be elaborated on below.

Before addressing samples and psychometric quality, however, it is important to consider that the existing instruments measure quite different aspects of parent–child communication, ranging from communication problems, openness or conformity and conversation orientation over problem-solving oriented, or avoidant communication to meta-communication. This variety reflects the complexity of communication and points to the fact that doing research on communication also should entail conceptual consideration. In addition to using measures with adequate psychometric properties, researchers and/or clinicians should be aware that they can aim at measuring quite different aspects of parent–child communication.

In terms of theoretical foundation, the information on most instruments was scarce. Considering that multiple studies were excluded due to using single-item measures (e.g., how would you rate the communication between you and your father/mother) or topic-specific measures (e.g., about alcohol, drugs, sex), there seems to be relatively little theory-based consensus in the field concerning how to conceptualize “parent–child communication” in a child-rated instrument. The two most dominating dimensions concern how open and/or problematic the communication is considered. However, the range of existing subscales indicates that there may be additional relevant dimensions of parent–child communication. In addition, the theoretical background of openness and communication problems also needs further clarification, since, for example, openness can be considered as a matter of openness for different perspectives or as openness for expressing emotions. Hence, a synthesis of previous models of parent–child communication, as has been attempted for the variable family communication (Murphy et al., 2017), would be desirable. Up to now, the field lacks standards for measuring other dimensions of parent–child communication.

The samples used ranged from general community samples via at-risk samples to clinical samples, with a dominance for community samples. More than half the studies were conducted in the North Americas (USA and Canada), with an even number of European and Asian studies in “second place,” followed by only a handful of studies from Middle and South America or Australia, and only one study from Africa. Although the USA samples included a mix of White, Black, Asian, and Hispanic Americans, there is clearly a need to examine and validate parent–child communication measures cross-culturally. Only the PACS has been used in clinical, at-risk, and community samples. The COMPA, PACI, RFCP, PCCS (Loeber), and PCCS (McCarty) instruments have been used in at-risk and community samples. The PACS and PCCS (McCarty) instruments can detect the differences in the quality of parent–child communication between these samples.

In terms of psychometric quality, the evidence backing most of the scales was insufficient. Our evaluation of this was based both on original publications of the instruments, as well as the synthesized data across studies that have used the instruments. Generally, little is known about most instruments, and even the English version of the PACS reached only 11 of 27 possible points in our quality assessment based on information from 51 publications. Adequate evidence for psychometric core aspects such as test–retest reliability or factorial structure was found for less than a handful of instruments. Across the studies, construct and discriminative validity have hardly been evaluated in the included studies. In addition, little information was available regarding convergent validity with regard to other measures such as observer-based (Hadley et al., 2013) or parent-based reports (Hartos & Power, 2000a, 2000b). However, it is important to note that while our main overall goal was to assess the psychometric properties of parent–child communication measures, this was not the goal of the reviewed studies. Rather, most of the included studies considered various research questions, of which parent–child communication was one of the several. At best, the results of some of the individual studies can be interpreted as preliminary/emerging evidence for construct and discriminative validity for one measure in one sample. However, when considered combined in this review, overall systematic evidence for the psychometric properties of even widely used scales (e.g., the PACS) must be said to be severely lacking.

We also aimed to consider availability of the instruments. Various abbreviations for the different instruments were often used inconsistently in the literature. Further confusion is added since some of the instruments are based on each other: The PACS has been used as one of several sources for the PCCS (Loeber), which, in turn, was used for the PCCS (McCarty) and the PCCS (Krohn). It is also noteworthy that the original PCCS (McCarty) instrument (child version) contains six items from the PACS almost literally, only the grammar has been changed from first to second person. The parent communication subscale of the PCCS (McCarty) consists of five items, with four from the PACS (three from the open, one from the problem subscale). The child communication subscale comprises three items with only one from the PACS. However, the two remaining items are fairly close to other PACS items. This leads to confusion about the measures and underlines the importance of indicating correct sources, number of items, scale range, and example items. In terms of availability, the instruments had been translated and used in a total of twelve languages.

## Limitations

To our knowledge, this review is the first to give a comprehensive and systematic summary of parent–child communication measures and their psychometric properties. However, there are some limitations. The review did not include observational or parent report measures. The selection criteria limited the search down to published peer-review articles but did not consider other publications, unless they were cited as a source for psychometric information in the included papers. In that case, they were considered in the quality assessment. If publications such as dissertations would have been included, other information regarding conceptual considerations and psychometric evidence might have been found. In terms of age, our search was limited to ages 8–21 years. Hence, the psychometric evidence for single instruments may differ with regard to emerging adults. Even though we searched for instruments in any language, we only considered publications in English, leading to a certain bias between English and non-English instruments. For that reason, the quality assessment does not allow for an exact comparison between instruments of different languages. Last but not least, since genuine psychometric studies were hardly found, we decided to not use the EMPRO tool (Valderas et al., 2008) to conduct the quality assessment as initially planned. Instead, we decided to gather psychometric information with an adapted tool that was more apt to also consider information found in a huge number of non-psychometric study reports.

## Implications and Conclusions

This review has implications for practice and research. For researchers and practitioners interested in examining parent–child communication from the child’s perspective, there are several child-rated instruments to choose from in English, Chinese, and some European languages. We have provided availability information for the four most-used child-rated parent–child communication scales in the methods section. The most widely used scale is the PACS (Barnes & Olson, 1982), which considers the degree of openness and problems in communication, and with some evidence of factor structure and other psychometric properties. However, the PACS should not necessarily be the default choice due to frequency alone. Upon choosing which instrument to apply, practitioners and researchers should take active and informed choices about which aspect of parent–child communication they aim to assess, as the instruments focus on different dimensions of parent–child communication. For example, whereas the PACS concerns openness and communication problems, the RFCP measures conformity and conversation orientation.

In terms of research implications, there is a need for more studies assessing the psychometric properties of parent–child communication scales, and when choosing an instrument, also conceptual and cross-cultural aspects should be carefully considered. To avoid confusion, instruments should be reported with correct sources, number of items, scale range, and example items. Future research should also consider triangulating child and parent reports as well as observational measures.

## Supplementary Information

Below is the link to the electronic supplementary material.

Supplementary file1 (DOCX 14 kb)

## Data Availability

No new data were created during the study.
