# Supplementary material for: A Systematic Review of Parent–Child Communication Measures: Instruments and Their Psychometric Properties
Source: Clin Child Fam Psychol Rev. 2022 Sep 27;26(1):121–42. doi: 10.1007/s10567-022-00414-3 (PMC9879831; doi:10.1007/s10567-022-00414-3)
Supplement: Supplementary file 1 — Supplementary file1 (DOCX 14 kb) [file 10567_2022_414_MOESM1_ESM.docx]

**Supplementary Table 1**

*Quality Assessment Guidelines*

| **Criterion** | Adequate (1) | Good (2) | Excellent (3) |
| --- | --- | --- | --- |
| Norms | M and SD for total score (and subscores if relevant) from a large sample (clinical > 100 or community > 400) | M and SD for total score (and subscores if relevant) from multiple large, relevant samples, at least one clinical and one nonclinical | Same as “good,” but must be from representative sample (i.e., random sampling, or matching to census data) |
| Internal Consistency (Cronbach's alpha, split half, etc.) | Median alpha value of 0.70–0.79 | Median alpha 0.80–0.89 | Median alpha ≥ 0.90 |
| Test-retest reliability (stability) | Most evidence shows test–retest correlations ≥0.70 over period of several days or weeks | Most evidence shows test–retest correlations ≥0.70 over period of several months | Most evidence shows test–retest correlations ≥0.70 over 1 year or longer |
| Content validity | Test developers clearly defined domain and ensured representation of entire set of facets | Same as “adequate,” plus all elements (items, instructions) evaluated by judges (experts or pilot participants) | Same as “good,” plus multiple groups of judges and quantitative ratings |
| Construct validity (e.g., predictive, concurrent, convergent, and discriminant validity) | Some independently replicated evidence of construct validity | Bulk of independently replicated evidence shows multiple aspects of construct validity | Same as “good,” plus evidence of incremental validity with respect to other clinical data |
| Dimensionality/factorial structure | Some evidence for factorial structure (e.g., one exploratory factor analysis) | Most evidence supports factorial structure (e.g., confirmatory factor analysis) | Most evidence supports factorial structure (e.g., several confirmatory factor analyses) |
| Discriminative validity | Small effect between a clinical and a non-clinical sample | At least medium effect between a clinical and a non-clinical sample | Same as “good,” but in more than one study |
| Validity generaliziation | Some evidence supports use with either more than one specific demographic group or in more than one setting | Bulk of evidence supports use with either more than one specific demographic group or in multiple settings | Bulk of evidence supports use with either more than one specific demographic group AND in multiple settings |
| Treatment sensitivity | Some evidence of sensitivity to change over course of treatment | Independent replications show evidence of sensitivity to change over course of treatment | Same as “good,” plus sensitive to change across different types of treatments |

***Note***. All criteria could be rated “no information available” and were rated “poor” (0) if less than “adequate”.
